# Treatment efficacy prediction model for patients with concurrent allergic rhinitis and laryngopharyngeal reflux

**DOI:** 10.3389/fmed.2026.1766580

**Published:** 2026-02-06

**Authors:** Pan Xie, Fusen Peng, Zhichao Xiao, Ying Zhang

**Affiliations:** Department of Otorhinolaryngology Head and Neck Surgery, Loudi Central Hospital, Loudi, China

**Keywords:** allergic rhinitis, laryngopharyngeal reflux, nomogram model, predictive analysis, treatment efficacy

## Abstract

**Objective:**

To construct and validate a prediction model based on the treatment efficacy of patients with laryngopharyngeal reflux (LPR) and allergic rhinitis (AR), and to provide a quantitative tool for individualized treatment decision-making.

**Methods:**

A total of 106 AR patients admitted to the hospital from January 2023 to November 2024 were included. They were randomly divided into a training set (*n* = 73) and a validation set (*n* = 33) at a ratio of 7:3. Independent influencing factors were screened by univariate and multivariate Logistic regression to construct a Nomogram model. The model performance was evaluated using receiver operating characteristic (ROC) curve, calibration curve, and decision curve analysis (DCA).

**Results:**

The baseline data of the training set and the validation set were balanced (*p* > 0.05). Multivariate analysis showed that BMI, duration of AR, number of allergens, and total serum IgE were independent predictors of treatment efficacy (*p* < 0.05). The areas under the ROC curve (AUC) were 0.809 (95% CI: 0.693–0.924) and 0.823 (95% CI: 0.630–1.000) respectively in the training set and validation set, and the calibration curve showed a good fit.

**Conclusion:**

The constructed Nomogram model can effectively predict the treatment response of patients with AR complicated by LPR and guide clinical stratified intervention.

## Introduction

Allergic rhinitis (AR) and laryngopharyngeal reflux (LPR) are common chronic diseases in otolaryngology, with global prevalence rates of 10%–40% and 10%–20%, respectively. The prevalence of AR among adults in China is 17.6, and 30%–50% of them are complicated with LPR. The co-occurrence of AR and LPR creates a substantial disease burden that significantly impacts quality of life, with overlapping symptoms including nasal congestion, throat irritation, chronic cough, and voice changes that often lead to misdiagnosis and delayed treatment (1). This comorbidity results in increased healthcare utilization, prolonged symptom duration, and substantial economic costs due to frequent medical visits, multiple diagnostic procedures, and polypharmacy approaches.

Currently, there are key challenges in clinical treatment: Approximately 30%–50% of AR patients complicated with LPR respond poorly to standard therapies (such as nasal corticosteroids combined with proton pump inhibitors), and there is significant heterogeneity in therapeutic efficacy. This difference may be related to factors such as BMI, allergy burden, and immune status. Therefore, it is urgent to construct a quantitative model to predict individualized treatment responses. The latest mechanism research reveals that the two share a Th2-type immune microenvironment. An increase in serum total IgE not only indicates AR but also enhances reflux sensitivity through the mast cell-esophageal mucosa axis (2, 3).

There are also major challenges in clinical diagnosis: The misdiagnosis rate of existing questionnaires and single indicators (such as serum IgE and esophageal pH monitoring) reaches 40%, and there is a lack of multi-dimensional assessment tools. This study intends to construct a Nomogram prediction model for treatment efficacy based on AR cohort by integrating clinical symptoms and immune indicators (IgE, eosinophil count) (3, 4). This visual tool can quantify individual differences in treatment efficacy, achieve one-stop clinical assessment, and reveal key driving factors through weight analysis, providing a basis for stratified intervention. This study may contribute to addressing knowledge gaps in predicting AR complicated with LPR and could potentially inform the development of more precise diagnostic and therapeutic approaches for allergic diseases.

## Materials and methods

### Study population

This retrospective study enrolled 106 consecutive patients diagnosed with AR complicated by LPR who were hospitalized at our institution between January 2023 and November 2024. All eligible patients were randomly divided into a training set (*n* = 73) and a validation set (*n* = 33) at a 7:3 ratio after data collection: the training set was used for model construction by screening independent predictive factors, and the validation set was used for model performance verification (to avoid overfitting). The enrollment period was consistent for both sets, with no separate time windows for enrollment. Patient selection was based on the following criteria: Inclusion criteria: (1) Clinical diagnosis of AR according to the Guidelines for the Diagnosis and Treatment of Allergic Rhinitis (2022) (5); (2) Concurrent diagnosis of LPR based on characteristic clinical symptoms including hoarseness, chronic throat clearing, globus sensation, and chronic cough; (3) Age ≥18 years; (4) Complete medical records available. Exclusion criteria: (1) Severe comorbid gastrointestinal disorders; (2) History of acid-suppressive medication use or anti-reflux surgery within the preceding 3 months; (3) Pregnant or lactating women; (4) Incomplete medical records; (5) Acute infections (within 2 weeks) or parasitic infestation history; (6) Exacerbations of other atopic diseases (e.g., asthma acute attack) within 1 month; (7) Systemic corticosteroid use within 3 months or immunotherapy/biologics use within 6 months; (8) Clinical history or physical examination suggestive of eosinophilic esophagitis (EoE), such as dysphagia or food impaction. Key characteristics of the study population (*n* = 106) were as follows: mean age 41.6 ± 9.4 years, male/female ratio 53/53 (50.0%/50.0%), mean BMI 22.9 ± 3.5 kg/m^2^, mean AR duration 8.2 ± 3.0 years, mean number of positive allergens 3.2 ± 1.1 kinds, mean total serum IgE 299.7 ± 101.5 IU/mL; 31.1% (33/106) of patients had comorbid asthma, and 51.0% (54/106) had comorbid sinusitis. The study was approved by the Ethics Committee of Loudi Central Hospital (Approval No. LDCH-25). Written informed consent was obtained from all participants prior to enrollment. Patient confidentiality was maintained throughout the study period, and all data were de-identified for analysis purposes. Clinical trial number: not applicable.

### Data collection

All data were systematically extracted from the hospital’s electronic medical record (EMR) system using standardized data collection protocols. The following variables were retrieved and documented: demographic characteristics including age, gender, and BMI; lifestyle factors encompassing smoking history, alcohol consumption history, and dietary habits (frequency of high-salt diet consumption: defined as consuming meals with added salt ≥5 g per meal (consistent with WHO’s high salt intake definition) for ≥5 times per week, assessed via self-reported dietary questionnaires); clinical parameters including duration of AR symptoms, presence of nasal polyps, history of comorbid asthma or sinusitis, and occupational exposure history; laboratory findings including total serum IgE levels (measured by chemiluminescence immunoassay within 24 h of admission, before treatment initiation) and percentage of peripheral blood eosinophils; patients with recent changes in allergen exposure (assessed via occupational exposure history and skin prick tests) were recorded and adjusted for in statistical analysis; allergy assessment data including family history of allergic diseases and number of positive allergens identified through standardized skin prick tests; and behavioral factors such as predominant sleep position (percentage of time spent in supine position during sleep, determined by self-reported 7-day sleep diaries before admission, calculated as the ratio of supine sleep time to total sleep time).

### Treatment regimens

All patients received standardized combined treatment for AR and LPR based on clinical guidelines. For AR management: intranasal corticosteroids (mometasone furoate nasal spray, two sprays per nostril, once daily) combined with oral antihistamines (loratadine, 10 mg once daily). For LPR management: proton pump inhibitors (esomeprazole, 40 mg twice daily, administered 30 min before breakfast and dinner). The total duration of treatment was uniformly 12 weeks for all patients, and the regimen was selected according to the Guidelines for the Diagnosis and Treatment of Allergic Rhinitis (2022) (6) and clinical practice guidelines for LPR, with no individualized dose adjustments except for documented contraindications.

### Outcome measures

The primary outcome measure was treatment efficacy, defined by the therapeutic response of laryngopharyngeal reflux (LPR) symptoms and assessed using the Reflux Symptom Index (RSI) score, a validated reflux-specific tool. The RSI evaluates core LPR symptoms including hoarseness, chronic throat clearing, globus sensation, and chronic cough. Treatment response was defined as follows: effective treatment was a reduction of ≥50% in RSI score compared to baseline, while ineffective treatment was a reduction of <50% in RSI score from baseline.

### Statistical analysis

Data analysis was performed using SPSS 25.0 and R 4.0.3 software. Measurement data were expressed as mean ± standard deviation. Independent-samples *t*-test was used for comparison between two groups, and analysis of variance was used for comparison among multiple groups. Count data were expressed as the number of cases and percentages (*n*, %). The chi-square test was used for comparison between groups. Logistic regression analysis was performed with treatment efficacy (effective = 1, ineffective = 0) as the dependent variable to screen independent predictive factors affecting the treatment efficacy of patients with AR complicated by LPR. A nomogram prediction model was constructed using R software based on independent influencing factors. The predictive efficacy of the model was evaluated by the Receiver Operating Characteristic (ROC) curve, and the Area Under Curve (AUC) and its 95% Confidence Interval (CI) were calculated. The calibration curve was used to evaluate the consistency between the predicted values and the actual observed values of the model. Decision curve analysis (DCA) was employed to assess the clinical utility of the nomogram by quantifying the net benefit across a range of threshold probabilities. *p* < 0.05 was considered statistically significant.

## Results

### Comparison of baseline data of patients in the training set and the validation set

No significant differences were found in indicators such as age, gender, smoking history, drinking history, family history of allergy, duration of AR, history of nasal polyps, and number of allergens between the training set (*n* = 73) and the validation set (*n* = 33) (*p* > 0.05), indicating comparability (Table 1).

**Table 1:** Comparison of baseline data of patients in the training set and the validation set.

Indicators	Training set (*n* = 73)	Validation set (*n* = 33)	*t/χ^2^*	*p*
Age (years)	42.21 ± 8.99	40.22 ± 10.20	1.012	0.314
Sex (male/female)	38/35 (52.05/47.95)	15/18 (45.45/54.55)	0.396	0.529
BMI (kg/m^2^)	23.16 ± 3.13	22.26 ± 4.21	1.226	0.228
Smoking history (yes/no)	27/46 (36.98/63.02)	10/23 (30.30/69.70)	0.447	0.503
Drinking history (yes/no)	13/60 (17.80/82.20)	3/30 (9.09/90.91)	0.753	0.386
Family history of allergy (yes/no)	35/38 (47.95/52.05)	12/21 (36.36/63.64)	1.235	0.266
Duration of AR (years)	8.12 ± 3.12	8.33 ± 2.98	0.325	0.746
History of nasal polyps (yes/no)	32/41 (43.84/56.16)	15/18 (45.45/54.55)	0.024	0.876
Number of allergens (kinds)	3.32 ± 1.12	3.11 ± 1.08	0.904	0.368
Total serum IgE (IU/mL)	300.31 ± 102.51	298.26 ± 99.89	0.096	0.923
Comorbid asthma (yes/no)	23/50 (315.51/68.49)	10/23 (30.30/69.70)	0.015	0.901
History of comorbid sinusitis (yes/no)	37/36(50.68/49.32)	17/16(51.51/48.49)	0.006	0.936
Occupational exposure (dust/chemicals)	20/53 (27.39/72.61)	10/23 (30.30/69.70)	0.095	0.758
Percentage of peripheral blood eosinophils (%)	10.86 ± 3.81	12.21 ± 4.21	1.635	0.105
Percentage of supine sleep (%)	68.99 ± 18.22	71.22 ± 19.23	0.574	0.567
Frequency of high-salt diet (≥5 times/week)	38/35 (52.05/47.95)	20/13 (60.60/39.40)	0.671	0.413

### Univariate analysis of treatment efficacy in patients of the training set

In the training set, significant differences were found between the ineffective group (*n* = 40) and the effective group (*n* = 33) in terms of indicators such as BMI, smoking history, duration of AR, number of allergens, total serum IgE, history of comorbid asthma/sinusitis, proportion of peripheral blood eosinophils, proportion of supine sleep, and frequency of high-salt diet (*p* < 0.05) (Table 2).

**Table 2:** Univariate analysis of treatment efficacy in patients of the training set.

Indicators	Ineffective group (*n* = 40)	Effective group (*n* = 33)	*t/χ^2^*	*p*
Age (years)	43.21 ± 8.92	41.82 ± 9.10	0.657	0.514
Sex (male/female)	21/19 (52.50/47.50)	17/16 (51.51/48.49)	0.007	0.933
BMI (kg/m^2^)	24.12 ± 3.33	22.21 ± 2.91	2.581	0.012
Smoking history (yes/no)	21/19 (52.50/47.50)	6/27 (18.18/81.82)	9.137	0.003
Drinking history (yes/no)	8/32 (20.00/80.00)	5/28 (15.15/84.85)	0.290	0.590
Family history of allergy (yes/no)	22/18 (55.00/45.00)	13/20 (39.39/60.61)	1.765	0.184
Duration of AR (years)	8.52 ± 3.21	6.82 ± 2.52	2.476	0.015
History of nasal polyps (yes/no)	22/18 (55.00/45.00)	10/23 (30.30/69.70)	4.479	0.034
Number of allergens (kinds)	3.51 ± 1.22	2.85 ± 1.01	2.483	0.015
Total serum IgE (IU/mL)	320.32 ± 108.52	260.80 ± 105.61	2.361	0.021
Comorbid asthma (yes/no)	18/22 (45.00/55.00)	5/28 (15.15/84.85)	7.465	0.006
History of comorbid sinusitis (yes/no)	25/15 (62.50/37.50)	12/21 (36.36/63.64)	4.942	0.026
Occupational exposure (dust/chemicals)	14/26 (35.00/65.00)	6/27 (18.18/81.82)	2.271	0.109
Percentage of peripheral blood eosinophils (%)	12.56 ± 3.84	8.23 ± 2.98	5.293	0.001
Percentage of supine sleep (%)	78.92 ± 15.21	52.61 ± 20.01	6.379	0.001
Frequency of high - salt diet (≥5 times/week)	25/15 (62.50/37.50)	13/20 (39.39/60.61)	3.868	0.049

### Multivariate logistic regression analysis

The treatment efficacy was taken as the dependent variable, and the variables with *p* < 0.05 in the univariate analysis were used as covariates. The variable assignments are shown in Table 3. Multivariate logistic regression was performed, and the results showed that BMI, the course of AR, the number of allergens, and total serum IgE were independent predictors of the treatment efficacy (*p* < 0.05) (Table 4).

**Table 3:** Variable assignment methods.

Variables	Meanings	Assignments
X1	BMI	Continuous variable
X2	Duration of AR	Continuous variable
X3	Number of allergens	Continuous variable
X4	Total serum IgE	Continuous variable
Y	Therapeutic effect	Ineffective = 1, Effective = 0

**Table 4:** Results of multivariate logistic regression analysis.

Item	B	SE	Wald	*p*	OR	95% CI
BMI	0.263	0.104	6.390	0.011	1.300	1.061–1.594
Duration of AR	0.267	0.107	6.195	0.013	1.306	1.058–1.612
Number of allergens	0.967	0.328	8.688	0.003	2.629	1.382–5.000
Total serum IgE	0.006	0.003	5.184	0.023	1.006	1.001–1.012

### Construction of the nomogram prediction model

**Figure 1:**
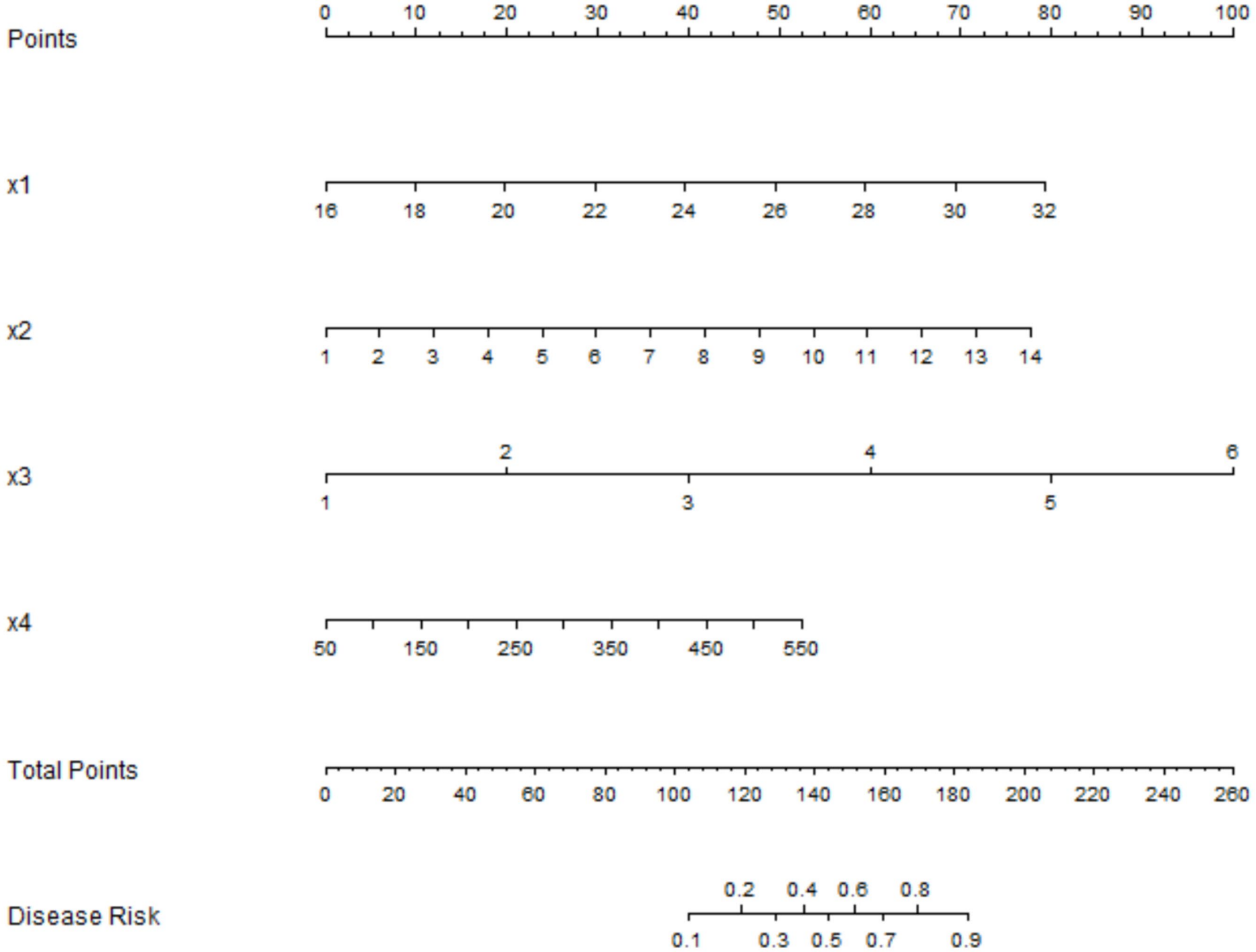
Nomogram prediction model for the treatment efficacy of patients with laryngopharyngeal reflux and allergic rhinitis. x1: BMI; x2: Duration of AR; x3: Number of allergens; x4: Total serum IgE.

Based on the independent influencing factors identified through multivariate Logistic regression analysis, a nomogram prediction model for the treatment efficacy of patients with laryngopharyngeal reflux and allergic rhinitis was constructed. Scores were assigned to each independent influencing factor in the model, and the total score for predicting the clinical efficacy was calculated, which was represented by the predicted probability (Figure 1).

### Evaluation and validation of the nomogram model

**Figure 2:**
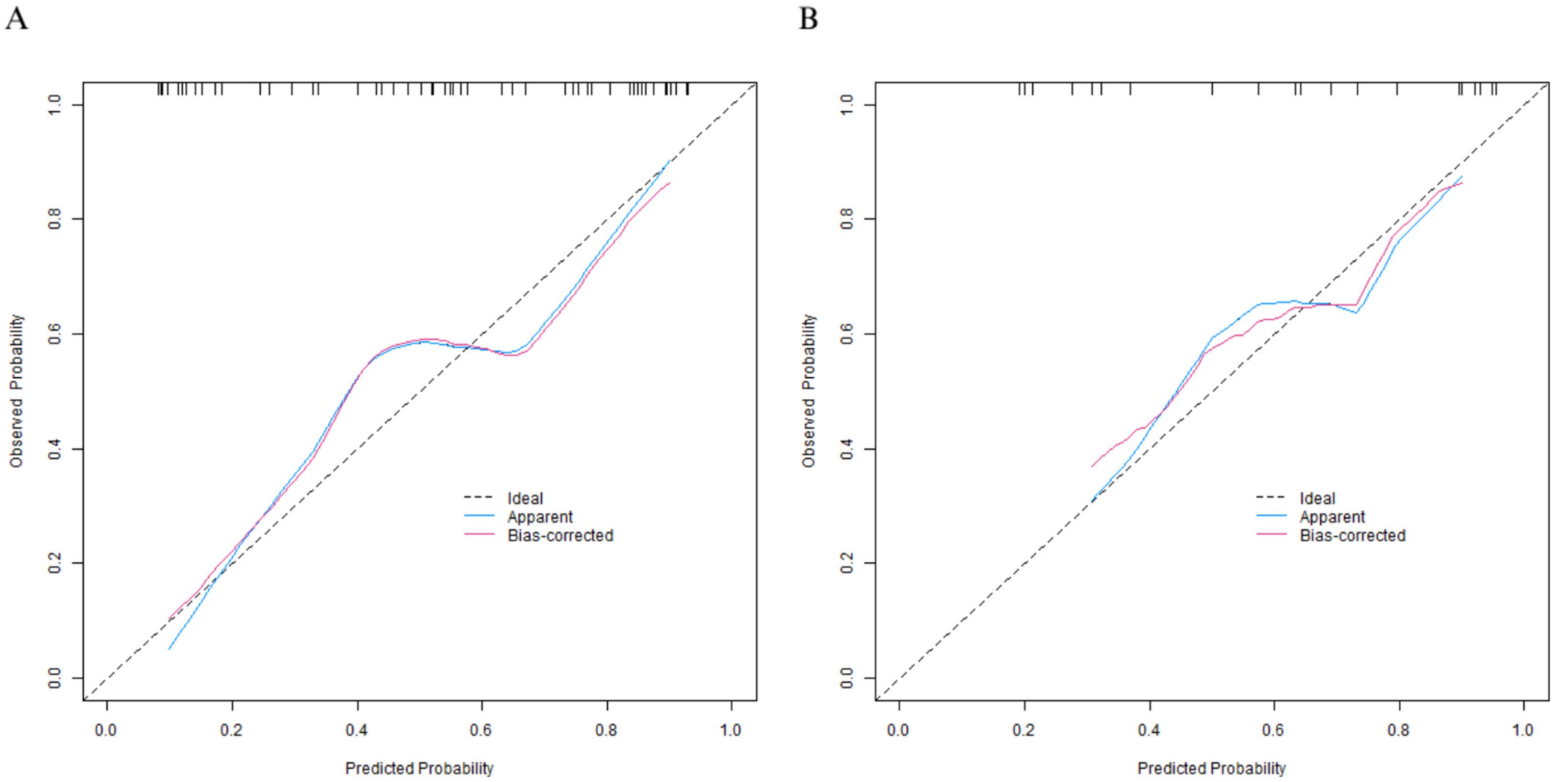
Calibration curves in the training set **(A)** and the validation set **(B)**.

**Figure 3:**
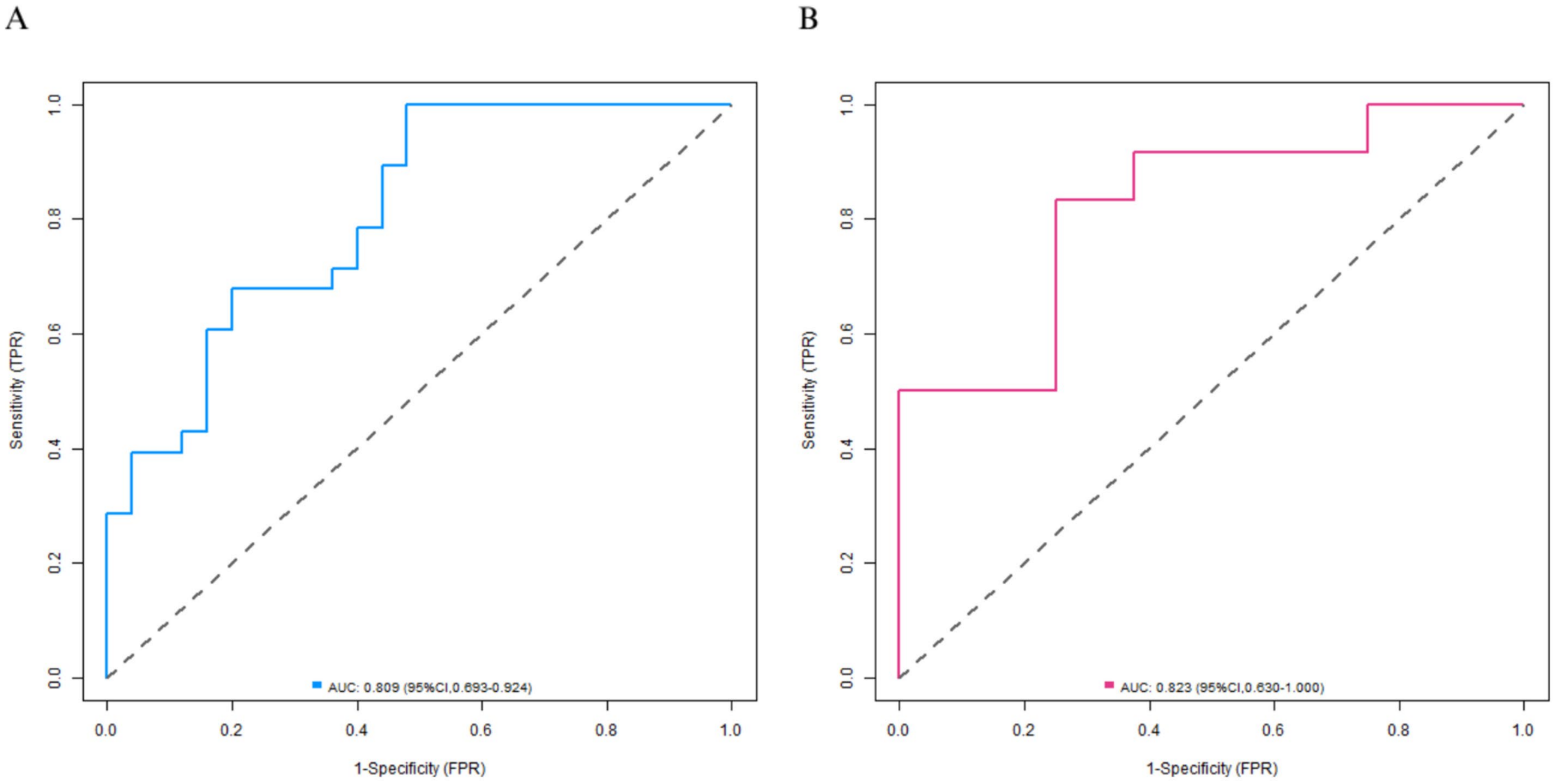
ROC curves in the training set **(A)** and the validation set **(B)**.

In the training set, the calibration curve showed that the mean absolute error between the predicted values and the actual values was 0.168. The Hosmer-Lemeshow test *p* = 0.104, indicating that the model was well-fitted. The ROC curve showed that the AUC was 0.809 (95% CI: 0.693–0.924), the sensitivity was 0.902, and the specificity was 0.520. The perfect sensitivity observed in the training set suggests that the model may have overfitted the training data, which warrants caution in interpretation. In the validation set, the mean absolute error was 0.182, the Hosmer–Lemeshow test *p* = 0.176, the AUC was 0.823 (95% CI: 0.630–1.000), the sensitivity was 0.833, and the specificity was 0.625. The calibration curve and the ROC curve are shown in Figures 2, 3, respectively.

### Decision curve analysis

**Figure 4:**
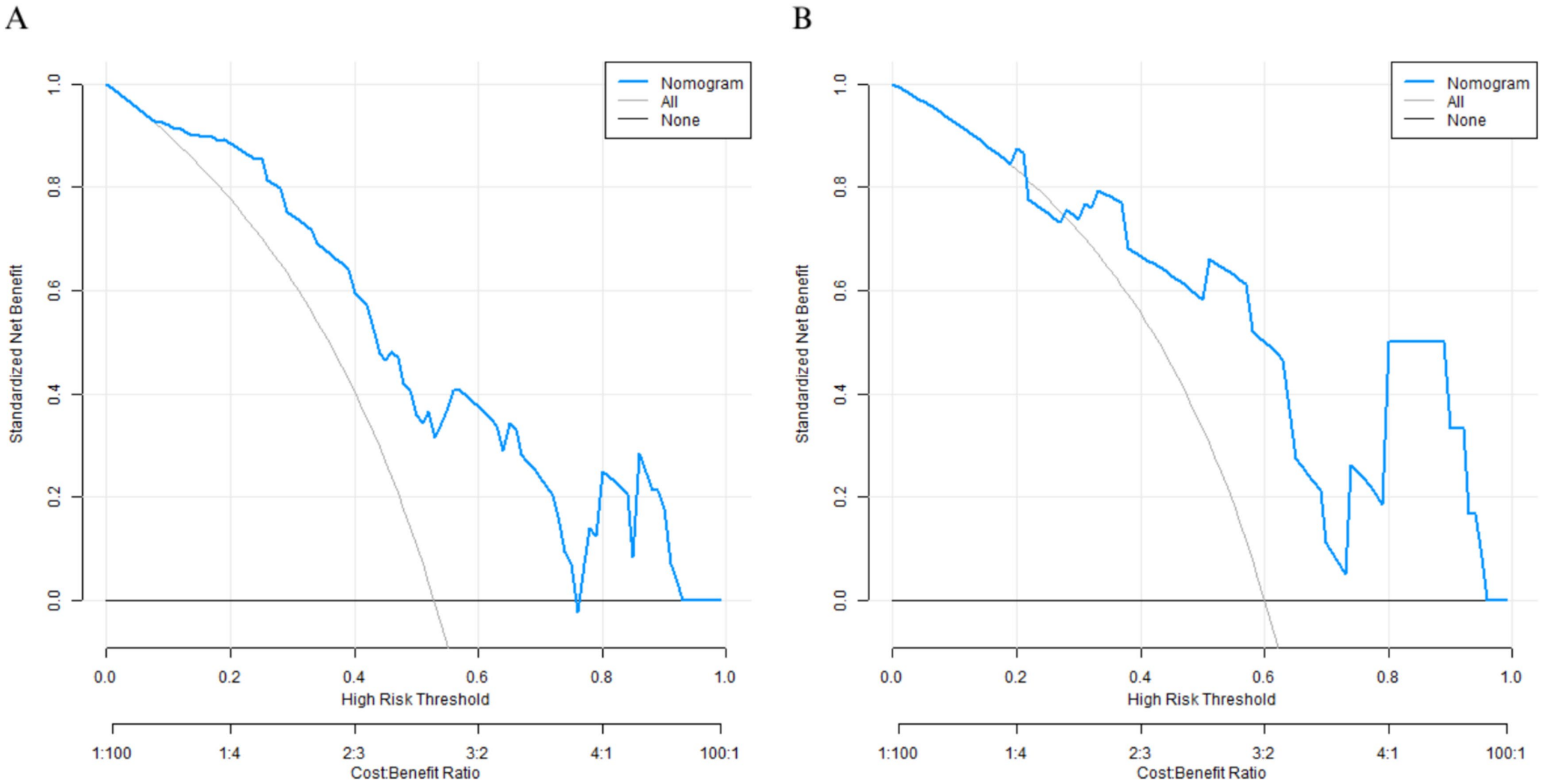
Decision curves in the training set **(A)** and the validation set **(B)**.

The model shows comparative net clinical benefit approximately between 8% and 78% threshold probabilities, corresponding to theoretical cost-benefit ratios ranging from 1:9 to 4:1. This broad range indicates potential clinical applicability across various risk tolerance levels and clinical scenarios. The validation set maintains similar net benefit patterns to the training set, which supports the model’s clinical robustness; however, conclusions regarding minimal overfitting are based on discrimination (AUC) and calibration metrics (calibration curve, Hosmer–Lemeshow test) rather than decision curve analysis alone (Figure 4).

## Discussion

Current clinical diagnosis for the concurrent presentation of allergic rhinitis (AR) and laryngopharyngeal reflux (LPR) faces several dilemmas. Traditional assessments rely heavily on subjective symptom questionnaires, and reliance on single indicators (e.g., serum IgE, esophageal pH monitoring) is associated with a misdiagnosis rate as high as 40%, highlighting a lack of integrated, multi-dimensional assessment tools. While the precise synergistic mechanisms between AR and LPR remain to be fully elucidated, existing evidence suggests interactive pathways, including: (1) Nasal congestion caused by AR forces patients to breathe through the mouth for a long time, weakening the function of the upper esophageal sphincter and increasing the risk of reflux; (2) Inflammatory mediators such as histamine and leukotrienes from post-nasal drip directly stimulate the pharyngeal mucosa, inducing local inflammation; (3) Excessive activation of the vagus nerve can simultaneously lead to hyperreactivity of the nasal mucosa and relaxation of the lower esophageal sphincter, forming an inflammation-reflux vicious cycle. The latest research on immune mechanisms has found that AR and LPR share a Th2-type immune microenvironment. An increase in total serum IgE is not only a marker of AR but also can enhance reflux sensitivity through the mast cell-esophageal mucosa axis (7). Therefore, constructing a prediction model for the treatment efficacy of patients with AR complicated with LPR can not only fill the gap in current diagnostic tools but also achieve the goal of precision medicine of early identification and early intervention by quantifying individual efficacy differences, providing a scientific basis for clinical stratified management.

In this study, based on a cohort of 106 AR patients, a Nomogram prediction model for the treatment efficacy of patients with AR complicated with LPR was constructed and validated by randomly dividing the training set (73 cases) and the validation set (33 cases) at a ratio of 7:3. The model construction process followed strict statistical procedures: First, 11 potentially relevant factors (BMI, smoking history, AR course, etc.) were screened out through univariate Logistic regression, and further multivariate analysis determined that BMI, AR course, the number of allergens and total serum IgE were independent influencing factors (*p* < 0.05). This screening process excluded the interference of confounding factors, ensuring the scientific nature of the model variables. The AUC in the training set and the validation set was 0.809 and 0.823 respectively, and the lower limits of the 95% confidence intervals were both > 0.6, indicating that the model had strong stability in different samples. The calibration curve showed that the predicted values fitted well with the actual observed values. The mean absolute errors in the training set and the validation set were 0.168 and 0.182 respectively, and the *p* values of the Hosmer–Lemeshow test were both > 0.05, further supporting the reliability of the model. Decision curve analysis showed that when the threshold probability was between 0.08 and 0.78, the net benefit of the model was significantly better than the “treat all” or “treat none” strategies, suggesting its clinical practicability. The visual feature of the Nomogram model is its significant advantage. By assigning weights to each independent influencing factor, clinicians can quickly calculate the probability of individual efficacy differences, achieving one-stop assessment. For example, for a patient with a BMI of 25 kg/m^2^, an AR course of 10 years, 4 types of allergens, and a total serum IgE of 400 IU/mL, the predicted probability of ineffective treatment is about 75%, suggesting that early anti-reflux treatment (such as proton pump inhibitors) should be considered and the AR management plan should be re-evaluated (in combination with validated symptom scores such as TNSS, disease control status, and current allergen exposure) (8). It should be emphasized that the model is a supportive tool for clinical decision-making, and adjustments to nasal corticosteroid doses should be guided by comprehensive clinical assessment rather than the model alone. This quantitative assessment mode helps to break the limitations of traditional empirical medicine and promote the precision diagnosis and treatment of AR-LPR comorbidity.

This study showed that for every 1-unit increase in BMI, the risk of ineffective treatment increased by 30% (OR = 1.300, 95% CI: 1.061–1.594). This result is consistent with the classic theory that obesity causes reflux: an increase in intra-abdominal pressure in obese individuals can directly lead to an imbalance in the pressure of the lower esophageal sphincter, and inflammatory factors (such as TNF-α and IL-6) secreted by adipose tissue may exacerbate the sensitivity of the esophageal mucosa (9). In addition, obese patients often have comorbid sleep apnea, and the proportion of supine sleep increases (in this study, the proportion of supine sleep in the ineffective group was 78.92%), further promoting nocturnal reflux events (10). In clinical practice, weight control should be used as a basic intervention measure for AR patients to prevent LPR (11). For every 1-year extension of the AR course, the risk of ineffective treatment increased by 30.6% (OR = 1.306). Long-term nasal mucosal inflammation can continuously damage the upper esophageal mucosa through the post-nasal drip-pharyngeal stimulation pathway, and the chronic inflammatory state may induce vagus nerve remodeling, reducing the anti - reflux barrier function of the esophagus (12). In this study, the average AR course in the ineffective group was 8.52 years, significantly longer than that in the effective group (6.82 years), suggesting that early intervention in AR to shorten the course may block its pathological progression to LPR (13). For every 1-type increase in the number of allergens, the risk of ineffective treatment increased by 1.629 times (OR = 2.629), which reflects a positive correlation between the allergic load and reflux susceptibility (14). Exposure to multiple allergens can activate a stronger Th2-type immune response, leading to an increase in serum IgE and eosinophil infiltration, and then promoting LPR through the following pathways: (1) Inflammatory mediators (such as tryptase) directly damage the esophageal mucosal barrier; (2) Cytokines (such as IL - 13) released by immune cells upregulate the expression of TRPV1 ion channels in esophageal epithelial cells, enhancing the sensitivity to acid reflux (15). Clinically, for AR patients sensitized to multiple allergens, potential differences in LPR treatment efficacy should be vigilant (16). For every 1-IU/mL increase in total serum IgE, the risk of ineffective treatment increased by 0.6% (OR = 1.006), suggesting a potential role for IgE in the allergy-reflux interaction (17). IgE may indirectly influence esophageal mucosal sensitivity through mast cell activation and release of inflammatory mediators such as histamine (18). Furthermore, some studies suggest that immune mediators may affect esophageal smooth muscle function, though the precise mechanisms in reflux require further investigation (19). In this study, the mean serum IgE in the ineffective group was 320.32 IU/mL, 22.8% higher than that in the effective group, suggesting that monitoring IgE levels can be used as an important indicator for efficacy difference stratification. Acidic reflux substances directly damage the pharyngeal mucosa, triggering an inflammatory response and squamous metaplasia, and the pharyngeal mucosa of AR patients is in a hypersensitive state due to long-term inflammation, and its tolerance to acid stimulation is significantly reduced (20).

This study has the following limitations: First, the sample size was small (a total of 106 cases) and the data were from a single center, leading to moderate specificity (training set: 0.520; validation set: 0.625) and wide 95% confidence intervals for AUC (e.g., validation set 95% CI: 0.630–1.000). These factors may limit the external validity of the model, and external validation in multi-center, larger independent cohorts is required to confirm its generalizability across different geographical and ethnic populations; Second, non-acidic reflux monitoring (such as impedance-pH monitoring) was not included, which may have missed some LPR patients with non-acid reflux as the main symptom; In addition, long-term follow-up was not conducted, and the ability of the model to predict long-term efficacy differences in LPR is not clear. The main reason for not conducting external validation was that the sample collection time was short within the research period (from January 2023 to November 2024), and the amount of single-center data was limited, making it difficult to obtain an independent external cohort. In the future, multi-center, large-sample studies are needed to integrate multi-modal data such as impedance-pH monitoring and esophageal manometry, and include lifestyle questionnaires (such as diet structure and exercise habits) to further optimize the model efficacy. At the same time, a risk warning APP can be constructed based on the model to achieve dynamic risk assessment through real-time data collection (such as sleep posture recorded by a smart bracelet), promoting the intelligent and personalized management of AR-LPR comorbidity. This study did not include detailed treatment compliance data, which may have influenced the observed efficacy and the model’s predictions. Future studies should aim to collect such data to further refine predictive models. Additionally, we did not perform endoscopic or histopathological examinations (the gold standard for EoE diagnosis) to systematically rule out EoE, which may introduce residual confounding due to overlapping symptoms between EoE and LPR. Future studies should include endoscopic/histopathological data to further improve the accuracy of patient enrollment. Finally, the model predicts efficacy based on the improvement in LPR symptoms (RSI). Future research aiming to predict the global response in patients with concurrent AR and LPR should consider integrating validated, disease-specific outcome measures for both conditions.

In conclusion, the Nomogram model constructed in this study successfully built a visual prediction tool for the treatment efficacy of patients with AR complicated with LPR by integrating multi-dimensional indicators such as BMI, AR course, the number of allergens, total serum IgE, and esophageal pH monitoring. The model not only has good discrimination and calibration but also its visual features are more convenient for clinical application. The analysis of each independent factor reveals the pathological chain of obesity-inflammation-reflux, suggesting that patients with high BMI, long-term disease, multiple allergies, and high IgE levels should be strengthened in reflux monitoring and early intervention. Despite the limitations of sample size and external validation, this study still provides a new paradigm for the interdisciplinary management of naso-pharyngeal-esophageal axis diseases and is expected to promote the development of a precision diagnosis and treatment system for allergic diseases.

## Data Availability

The original contributions presented in the study are included in the article/supplementary material, further inquiries can be directed to the corresponding author.
